# Supplementary material for: Lung Fibrosis Is Improved by Extracellular Vesicles from IFNγ-Primed Mesenchymal Stromal Cells in Murine Systemic Sclerosis
Source: Cells. 2021 Oct 13;10(10):2727. doi: 10.3390/cells10102727 (PMC8535048; doi:10.3390/cells10102727)
Supplement: Supplementary file 1 [file cells-10-02727-s001.zip › cells-1409090-supplementary.pdf]

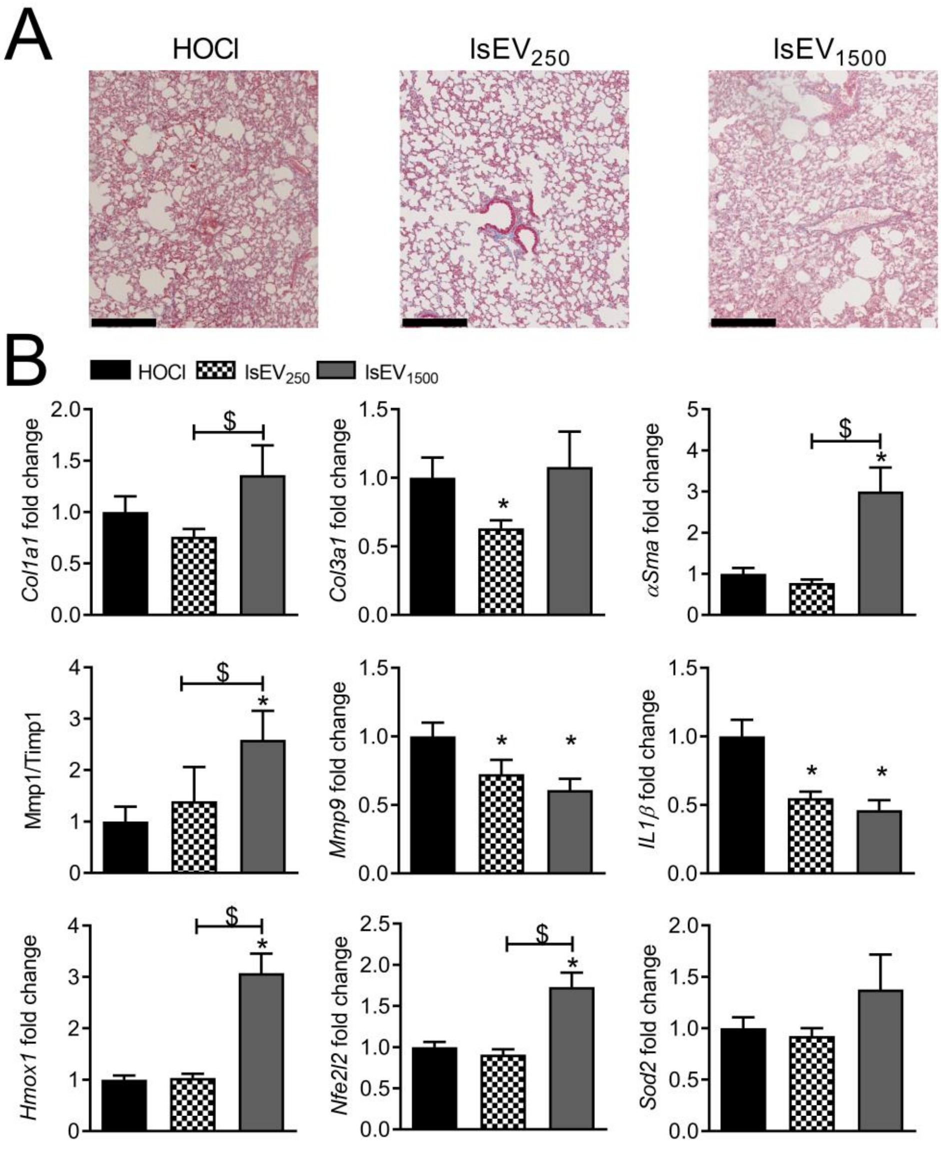

Supl Figure 1. Dose effect of lsEVs isolated from MSCs in the murine model of HOCl-induced SSc. **(A)** Photographs of representative histological sections of lungs after Masson's trichrome staining in control mice (HOCl) and mice that have been injected with 250 or 1500 ng of large size extracellular vesicles ($lsEV_{250}$ or $lsEV_{1500}$, respectively) (scale bar, 250 µm). **(B)** Gene expression in lung samples as expressed as fold change in treated versus HOCl control mice. Data are presented as mean ± SEM (n=8 to 12 per group; *: $p<0.05$ versus control or $: $p<0.05$ versus the indicated group of mice).
